# L-Malate Protects Skin Against Ultraviolet-Induced Ferro-Aging

**DOI:** 10.3390/metabo16100775

**Published:** 2026-10-13

**Authors:** Huanhuan Xu, Chuhui Song, Zhangqi Mao, Wenze Zhang, Hengyu Luo, Xiaoxuan Wu, Yingying Yu, Lili Huang, Liwen Zhang, Zuyong Zhang, Xuexian Fang

**Affiliations:** 1 Department of Nutrition and Toxicology, School of Public Health and Nursing, Hangzhou Normal University, Hangzhou 311121, China; 2 Department of Neurosurgery and Pharmacy, Hangzhou Third People’s Hospital, Hangzhou 310009, China; 3 Department of Cardiology, Sir Run Run Shaw Hospital, Zhejiang University School of Medicine, Hangzhou 310016, China; 4 Zhejiang Key Laboratory of Cardiovascular Intervention and Precision Medicine, Engineering Research Center for Cardiovascular Innovative Devices of Zhejiang Province, Hangzhou 310016, China; 5 Polytechnic Institute, Zhejiang University, Hangzhou 310015, China; 6 Zhejiang Key Laboratory of Medical Epigenetics, Hangzhou Normal University, Hangzhou 311121, China

**Keywords:** L-malate, ferro-aging, photoaging, malic enzyme, lipid peroxidation

## Abstract

**Background**: Chronic exposure to ultraviolet (UV) radiation, particularly UVB, is the primary driver of skin photoaging. Ferro-aging, a recently identified iron-triggered, lipid peroxidation-driven cellular senescence program distinct from acute ferroptotic cell death, has emerged as a critical contributor to age-related pathologies, yet its role in skin photoaging remains largely unexplored. **Methods**: In this study, we systematically evaluated the anti-photoaging efficacy of L-malate (LM), a naturally occurring organic acid, using both in vitro human skin cell models (HaCaT keratinocytes and HSFs) and an in vivo BALB/c nude mouse model of chronic UV irradiation. **Results**: Our results demonstrate that LM treatment significantly attenuated UVB-induced cellular senescence, DNA damage, and extracellular matrix remodeling in a dose-dependent manner. Mechanistically, LM may act by increasing the expression of malic enzymes (ME1 and ME2), thereby restoring intracellular NADPH levels and glutathione homeostasis. This metabolic restoration effectively blocks the ferro-aging cascade, as evidenced by suppressed ACSL4 upregulation, the maintenance of GPX4 protein levels, and reduced lipid peroxidation. Importantly, topical application of LM in UV-irradiated mice significantly ameliorated in vivo photoaging phenotypes and suppressed ferro-aging markers. **Conclusions**: In summary, our findings demonstrate that LM may inhibit ferro-aging through the ME1/ME2-NADPH antioxidant axis. This study reveals the possibility of ferro-aging as a potential novel therapeutic target in skin photoaging and suggests that LM is a promising naturally derived ferro-aging inhibitor with translational potential in anti-aging skincare.

## 1. Introduction

Skin, the largest organ of the human body, serves as the first line of defense against environmental insults, including solar radiation, pathogens, and chemical pollutants. Meanwhile, skin is also one of the most visually apparent sites of human aging. The thinning of the ozone layer caused by global air pollution has significantly increased human exposure to ultraviolet radiation [1]. Although ultraviolet (UV) radiation promotes the synthesis of essential pigments and vitamin D in the body, it can also induce skin carcinogenesis and photoaging [2]. Skin aging manifests as a progressive loss of structural integrity and physiological function, resulting in wrinkle formation, loss of elasticity, impaired barrier function, and delayed wound healing [3,4]. This process is driven by both intrinsic genetic factors and extrinsic environmental factors, among which chronic exposure to ultraviolet (UV) radiation, referred to as photoaging, is the predominant driver [5,6]. Unlike intrinsic genetic aging, environmentally induced aging can be delayed or mitigated through environmental modification and enhanced protection.

Within the UV spectrum, ultraviolet (UV) radiation can be classified into three bands: UVC (100–280 nm), UVB (280–320 nm), and UVA (320–400 nm) [7]. The UVB band (280–320 nm) exhibits the greatest biological activity. Its skin-damaging effect is 800–1000-fold higher than that of an equivalent dose of UVA, earning it the name “burning rays” [8]. UVB primarily penetrates the epidermal layer and promotes intrinsic aging along with wrinkle formation, pigmentation disorders, and photocarcinogenesis [9]. The central role of oxidative stress in photoaging has long been recognized, yet specific druggable pathways remain elusive [10,11]. Therefore, identifying bioactive and safe compounds that can protect skin cells from UVB-induced photoaging is of substantial research interest and therapeutic significance.

Iron is essential for life; this trace element is indispensable to nearly all organisms, and in its absence, cells are largely unable to acquire and utilize oxygen efficiently. However, this redox activity can drive the production of reactive oxygen species (ROS) and promote lipid peroxidation [12]. A notable accumulation of iron in the skin is believed to contribute to the acceleration of the skin aging process [13,14]. Dysregulation of iron homeostasis may also potentiate aging-related phenotypes and the senescence-associated secretory phenotype (SASP) [15,16]. Previous advances in aging research have focused on a novel form of regulated cell death termed ferroptosis, which is characterized by iron-dependent accumulation of lethal lipid peroxides [17,18,19]. More recently, Liu et al. formally defined ferro-aging as a conserved, iron-triggered, lipid peroxidation-driven cellular senescence program that is distinct from acute ferroptotic cell death and general oxidative stress [13]. Nevertheless, this nascent concept is currently confronted with multiple challenges and has yet to establish universally recognized diagnostic criteria. Despite its conceptual limitations, ferro-aging is increasingly supported by evidence identifying ACSL4 as a central effector linking iron overload, lipid peroxidation, and age-related cellular senescence [20]. Mechanistically, ferro-aging is orchestrated by the enzyme acyl-CoA synthetase long-chain family member 4 (ACSL4), which catalyzes the synthesis of polyunsaturated fatty acid (PUFA)-containing phospholipids, thereby rendering cells highly susceptible to iron-dependent lipid peroxidation [21,22]. In aged tissues, however, ACSL4 upregulation sustains non-lethal lipid peroxidation, thereby causing progressive cellular dysfunction and senescence [23]. However, the role of ferro-aging in skin photoaging remains largely unexplored.

L-malate (LM) is an endogenous small-molecule organic acid and a key intermediate in the tricarboxylic acid (TCA) cycle, with excellent biocompatibility and safety profiles [24]. It has long been recognized for its pivotal role in metabolic regulation and antioxidant function across diverse cell types and multiple organs [25,26,27,28]. Studies have shown that L-malic acid and malic enzyme-mediated metabolism critically regulate cellular senescence and may modulate senescent phenotypes and progression by affecting energy supply, oxidative stress, and mitochondrial function [29]. Specifically, LM is the primary substrate of malic enzymes (ME1 and ME2), which catalyze the oxidative decarboxylation to regenerate nicotinamide adenine dinucleotide phosphate (NADPH), a critical cofactor for maintaining the reduced glutathione (GSH) pool and glutathione peroxidase 4 (GPX4) activity, the master regulator of ferroptosis and ferro-aging [25]. Despite these promising properties, the protective effect of LM against skin photoaging and its potential association with the ferro-aging pathway have not been investigated.

In this study, we systematically evaluated the anti-photoaging efficacy of LM using UVB-irradiated human skin cell models in vitro and nude mouse models in vivo. Our results demonstrate for the first time that LM treatment effectively attenuates UVB-induced photoaging phenotypes. Mechanistically, we reveal that LM upregulates ME1 and ME2 expression, thereby restoring the NADPH pool and blocking the ferro-aging cascade. These findings suggest that ferro-aging may be a therapeutic target for photoaging of the skin and indicate that LM is a naturally derived ferro-aging inhibitor worthy of further study, with some translational potential for use in anti-aging skincare products and clinical interventions.

## 2. Materials and Methods

### 2.1. Cell Culture

HaCat and HSF cells were purchased from the Cell Bank of the Chinese Academy of Science (Shanghai, China) and confirmed via STR profiling. Cells were cultured in DMEM medium (Sangon Biotech, Shanghai, China) containing 10% heat-inactivated FBS (Sangon Biotech, Shanghai, China) and 1% penicillin–streptomycin (Meilunbio, Dalian, China) at 37 °C in a humidified atmosphere with 5% CO_2_. The culture medium was refreshed every 48 h, and cells were passaged upon reaching 90% confluence.

### 2.2. UVB Irradiation on the Cells

HaCaT or HSF cells were randomly divided into the following groups: control group, UVB group and UVB + LM group. HaCaT and HSF cells were exposed to UVB radiation using a UV irradiation system (VL-3000PRO, V-leader, Beijing, China). Cells in the control group were shielded from UV radiation using aluminum foil before exposure. Immediately after irradiation, cells were treated with the indicated concentrations of LM or left untreated (control), and cultured 24 h for subsequent analysis.

### 2.3. Animal Experiments

All experiments involving animals were performed in accordance with the National Institutes of Health’s Guide for the Care and Use of Laboratory Animals and were approved by the Animal Care and Use Committee of Hangzhou Normal University (NO. HSD-20260320-02). BALB/c nude mice (male, six-week-old) were purchased from GemPharmatech (Nanjing, China) and acclimatized for one week before the start of experiments. All mice were housed under specific pathogen-free (SPF) conditions with controlled temperature, 40–60% relative humidity, and a 12 h light/dark cycle, with free access to food and water.

Before the experiment commenced, mice were randomly assigned to the blank control group (*n* = 3), the UV group (*n* = 8), the UV + solvent group (*n* = 8), and the UV + LM group (*n* = 8). To establish a skin photoaging model, mice were irradiated with ultraviolet (UV) radiation every other day at the minimal erythema dose (MED). Before each irradiation, the lamps were warmed up for 30 min to ensure stable output prior to use. The irradiation dose was gradually increased as follows: UVA 10 mJ/cm^2^ and UVB 50 mJ/cm^2^ for the first two weeks, UVA 20 mJ/cm^2^ and UVB 100 mJ/cm^2^ during the third week, UVA 60 mJ/cm^2^ and UVB 150 mJ/cm^2^ in the fourth week, and UVA 80 mJ/cm^2^ and UVB 200 mJ/cm^2^ from the fifth to the eighth week, resulting in cumulative doses of 1 J/cm^2^ and 5 J/cm^2^, respectively.

After each irradiation session, 0.1 g of the designated formulation (blank vehicle, glycerol–petroleum jelly mixture or glycerol–petroleum jelly mixture containing 10% LM at pH 4.0–4.5) was applied topically to a 4 cm^2^ area centered on the spinal midline of the irradiated dorsal skin of mice, according to their experimental group assignment and left for 2h to allow absorption. Once the formulation was largely absorbed, the mice were returned to their individual cages and maintained for general health and skin condition.

### 2.4. Cell Viability Assay

HaCaT and HSF cells were counted and plated in 96-well plates at 1 × 10^4^ cells per well and incubated overnight. After 24 h, various concentrations of L-malic acid were added to UVB-exposed cells, and the cells were cultured for an additional 24 h. Then cell viability was assessed using the Cell Counting Kit-8 (CCK8; Meilunbio, Dalian, China) according to the manufacturer’s instructions. After incubation at 37 °C for 30–60 min, the optical density (OD) was measured at 450 nm using a microplate reader (Thermo Fisher Scientific, Shanghai, China).

### 2.5. SA-β-Gal Assay

SA-β-gal staining was performed by a commercial kit (C0602, Beyotime; Shanghai, China). Briefly, HaCaT and HSF cells were counted, seeded into 6-well plates at 2 × 10^5^ cells per well, incubated overnight, and treated accordingly. Cells were then washed with PBS, fixed at room temperature for 15 min, and incubated with SA-β-gal staining working solution at 37 °C for 16–18 h. Finally, images were captured on an inverted phase-contrast microscope (Olympus, Tokyo, Japan) and analyzed with ImageJ (version 1.38) software.

### 2.6. Lipid Peroxidation Assay

HaCaT and HSF cells were counted, seeded into 12-well plates at 1 × 10^5^ cells per well, and incubated overnight. The cells were then subjected to UV irradiation and/or LM treatment and cultured for an additional 24 h. The fluorescent probe C11-BODIPY (581/591) (D3861, Thermo Fisher Scientific, Waltham, MA, USA) was employed to monitor intracellular levels of lipid peroxidation. Subsequently, cells were treated with 5 µM BODIPY working solution and incubated in darkness at 37 °C for 30 min. Finally, samples were analyzed on a flow cytometer (Agilent Technologies, Santa Clara, CA, USA), and the data were processed using FlowJo (version 10.8.1) software.

### 2.7. FerroOrange Staining

Intracellular Fe^2+^ levels were measured using the fluorescent probe FerroOrange (F374, Dojindo, Kyushu, Japan). HACAT and HSF cells were seeded in 12-well plates. After treatment with different doses of UVB irradiation, the cells were washed twice with HBSS. The cells were then incubated in the dark for 30 min with a 1 μM FerroOrange working solution (diluted in HBSS) under culture conditions of 37 °C and 5% CO_2_. Finally, images were captured using an inverted fluorescence microscope (Nikon TS2R, Tokyo, Japan) and quantitative analysis was performed using ImageJ (version 1.38) software.

### 2.8. Measurement of Intracellular NADPH

Intracellular NADPH concentrations were measured using a commercial kit (S0175, Beyotime, Shanghai, China) according to the manufacturer’s instructions. HaCaT and HSF cells were counted and seeded into 6-well plates at 2 × 10^5^ cells per well, cultured overnight in an incubator, and subjected to the corresponding experimental treatments. The cells were then lysed with 200 µL of NADP^+^/NADPH extraction buffer, and the lysate was centrifuged at 12,000 rpm for 10 min at 4 °C to collect the supernatant, which was then divided into two aliquots. To eliminate the influence of NADP^+^, the 10-fold diluted supernatant was heated at 60 °C for 30 min. Subsequently, 100 µL of G6PDH mixture was added to each sample, followed by incubation at 37 °C for 10 min in the dark. Then, 10 µL of NADPH developer was added to each well, and the plate was incubated at 37 °C for 60 min. The data were acquired by measuring the optical density (OD) at 450 nm using a microplate reader. The NADPH concentration was calculated based on the standard curve and the protein concentration determined by the BCA assay.

### 2.9. Measurement of Intracellular GSH/GSSG

Total GSH and GSSG levels were measured using a commercial kit (S0053, Beyotime, Shanghai, China) according to the manufacturer’s instructions. Briefly, both cells and animal tissues were processed using a unified extraction protocol. HaCaT cells were seeded at 2 × 10^5^ cells per well in 6-well plates and cultured, followed by treatment with or without LM under UVB irradiation. Subsequently, cells were harvested by trypsinization and centrifuged to obtain cell pellets, whereas mouse skin samples were weighed, and 10 mg of tissue was retrieved from liquid nitrogen and homogenized using a grinder. Both cell pellets and tissue homogenates were processed identically: Protein Removal Reagent M solution was added to the cell pellets/skin tissue homogenates, followed immediately by vortexing. The samples were subjected to two rapid freeze–thaw cycles using liquid nitrogen and a 37 °C water bath. After incubation on ice for 10 min, the samples were centrifuged at 10,000 rpm for 10 min at 4 °C, and the supernatant was collected for the determination of glutathione (GSH) and oxidized glutathione (GSSG) levels.

### 2.10. Quantitative Real Time PCR

Total RNA was extracted from tissues or cells using FreeZol reagent (R711, Vazyme, Nanjing, China) and reverse-transcribed into cDNA with the Hifair^®^ II 1st Strand cDNA Synthesis SuperMix (11120 ES, Yeasen, Shanghai, China). Quantitative PCR was performed on a QuantStudio Real-Time PCR System (Life Technologies, Carlsbad, CA, USA) using SYBR Green Master Mix (11202 ES, Yeasen, Shanghai, China). Relative gene expression levels were determined by the 2^−ΔΔCt^ method and normalized to *GAPDH* mRNA as the internal reference.

### 2.11. Immunoblotting

Total protein was extracted from cells in RIPA buffer supplemented with protease inhibitors. Equal amounts of protein were separated by SDS-PAGE and transferred onto PVDF membranes (Millipore, Billerica, MA, USA), followed by blocking with 5% milk for 2 h. The membranes were probed with the following primary antibodies: anti-P16 (A4794, ABclonal, Wuhan, China), anti-P21 (A19094, ABclonal, Wuhan, China), anti-P53 (10442-1-AP, Proteintech), anti-γH2AX (AP1555, ABclonal), anti-Lamin B1 (A11459, ABclonal, Wuhan, China), anti-MMP1 (A22080, ABclonal, Wuhan, China), anti-MMP9 (A26079, ABclonal, Wuhan, China), anti-GPX4 (67763-1-Ig, Proteintech, Wuhan, China), anti-ACSL4 (A20414, ABclonal, Wuhan, China), anti-ME1 (ab97445, Abcam, Cambridge, UK), anti-ME2 (A9650, ABclonal, Wuhan, China), and anti-GAPDH (60004-1-Ig, Proteintech, Wuhan, China).

### 2.12. Measurement of Skin Elasticity by Pinch Test

To evaluate skin elasticity, a pinch test was performed on the dorsal midline skin. The skin was gently grasped between the thumb and forefinger and lifted while ensuring that the limbs of the mouse remained in contact with the ground. The skin was then immediately released, and the time required for the lifted skin to fully recoil to its original position was recorded in seconds.

### 2.13. Measurement of Skin Water Content

Fresh skin tissue (approximately 0.1 g) was precisely weighed to obtain the wet weight and then dried in an oven at 105 °C for 4 h. After drying, the sample was removed and the dry weight was accurately recorded. The skin water content was calculated using the following formula:

Skin water content (%) = [(wet weight − dry weight)/wet weight] × 100

### 2.14. Histological Analysis

Immediately after dissection, skin tissues were fixed in 4% paraformaldehyde. Fixed samples were dehydrated through a graded ethanol series, cleared in xylene, infiltrated with paraffin, and embedded. Paraffin sections were cut at 5 μm thickness. For hematoxylin and eosin (H&E) staining and Masson’s trichrome staining, sections were stained following standard protocols and examined under a light microscope.

### 2.15. Immunohistochemistry (IHC)

Paraffin-embedded skin sections were deparaffinized in xylene, rehydrated through a graded ethanol series, and subjected to heat-induced antigen retrieval in citrate buffer. Endogenous peroxidase activity was quenched with 3% hydrogen peroxide for 15 min at room temperature in the dark. After blocking with goat serum for 30 min, sections were incubated with primary antibodies (AB46545, AB97445, and A9650; ABclonal, Wuhan, China) overnight at 4 °C. Following PBS washes, sections were incubated with an HRP-conjugated secondary antibody (Thermo Fisher Scientific, Waltham, MA, USA) for 50 min at room temperature. Color was developed using a DAB substrate kit (Thermo Fisher Scientific, Waltham, MA, USA), and nuclei were counterstained with hematoxylin. Finally, sections were dehydrated, cleared in xylene, mounted, and examined under a light microscope.

### 2.16. Statistics

All data are expressed as mean ± standard error of the mean (SEM). Statistical analyses were performed using GraphPad Prism 9.0. The required sample size of each experiment was estimated by power calculations based on data from previous studies and preliminary experiments with an alpha level of 0.05 and 80% power. To avoid observer bias, the operators were blinded during experimental procedures involved in histology, quantitative PCR, and the measurement of GSH/GSSG. Comparisons between two groups were performed using a two-tailed unpaired Student’s *t*-test. For multiple-group comparisons, one-way analysis of variance (ANOVA) followed by Tukey’s post hoc test was applied. A *p* value < 0.05 was considered statistically significant.

## 3. Results

### 3.1. Establishment of UVB-Induced Photoaging Model in HaCaT Cells

**Figure 1:**
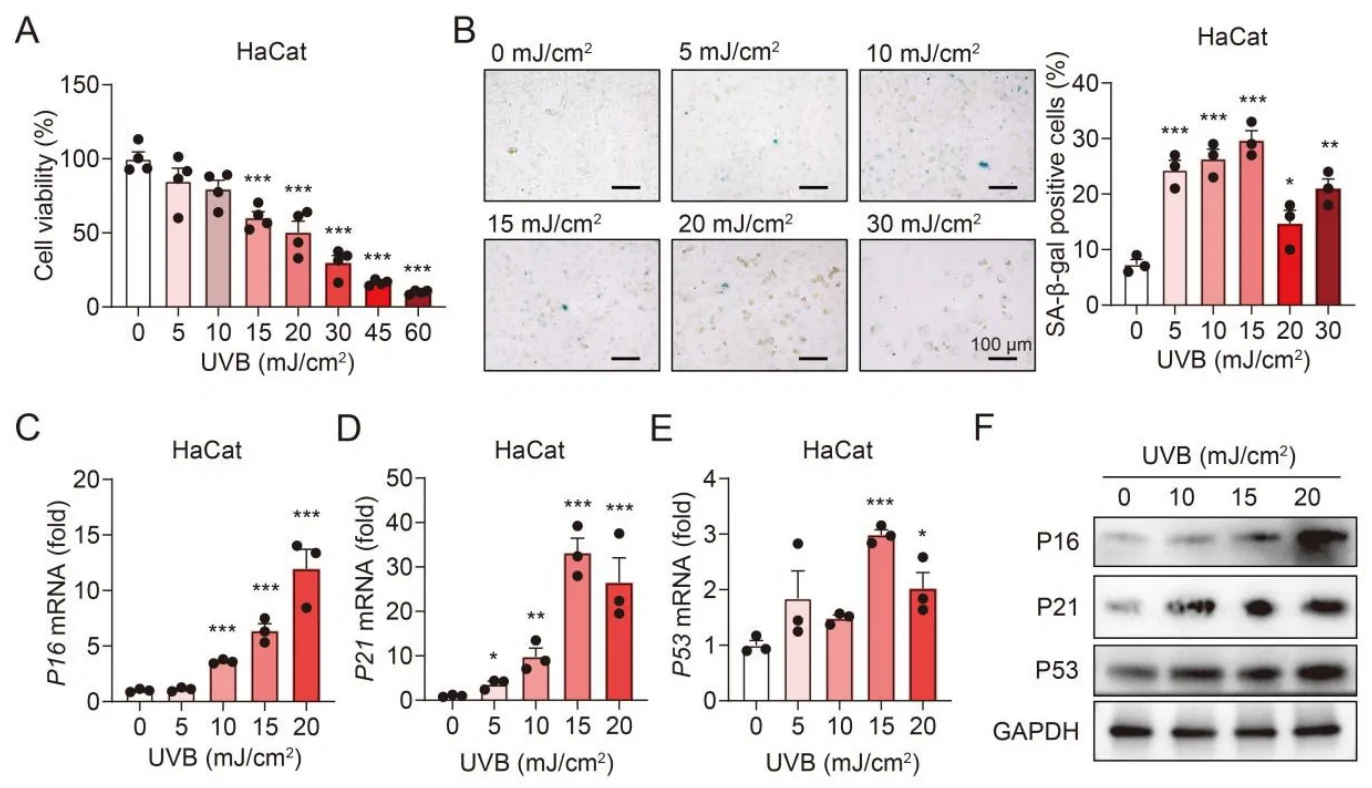
Establishment of UVB-induced photoaging model in HaCaT keratinocytes. (**A**) Cell viability of HaCaT cells exposed to gradient doses of UVB (0–60 mJ/cm^2^) (*n* = 4). (**B**) Representative images of SA-β-gal staining and quantitative analysis of SA-β-gal-positive cells in HaCaT cells after UVB irradiation (*n* = 3). (**C**–**E**) Relative mRNA expression levels of senescence markers *P16* (**C**), *P21* (**D**), and *P53* (**E**) in HaCaT cells treated with indicated UVB doses (*n* = 3). (**F**) Representative immunoblots of P16, P21, and P53 protein levels in HaCaT cells following exposure to indicated UVB doses. Data are presented as mean ± SEM. * *p* < 0.05, ** *p* < 0.01, and *** *p* < 0.001 versus control group.

To establish an in vitro model of skin photoaging, immortalized human keratinocytes (HaCaT cells) were exposed to a range of UVB doses. As shown in Figure 1A, UVB irradiation significantly reduced HaCaT cell viability in a dose-dependent manner. Senescence-associated β-galactosidase (SA-β-gal) staining revealed that a subset of UVB-irradiated HaCaT cells displayed characteristic blue positive staining, confirming the successful induction of premature senescence (Figure 1B). Semi-quantitative analysis further demonstrated that the most pronounced difference was observed at the dose of 15 mJ/cm^2^. To further characterize the molecular features of photoaging, we examined the expression of well-established senescence markers. UVB exposure dose-dependently upregulated *P16* mRNA levels (Figure 1C), whereas *P21* and *P53* mRNA expression peaked at approximately 15 mJ/cm^2^ (Figure 1D,E). Consistently, immunoblotting analysis demonstrated a dose-dependent increase in P16, P21, and P53 protein levels following UVB irradiation (Figure 1F). Collectively, these results demonstrate that UVB irradiation successfully induces senescence in HaCaT cells. Based on the integrated assessment, a dose of 15 mJ/cm^2^ was selected as the optimal irradiation dose for establishing the photoaging model in subsequent experiments.

### 3.2. LM Alleviates UVB-Induced Senescence and DNA Damage in HaCaT Cells

**Figure 2:**
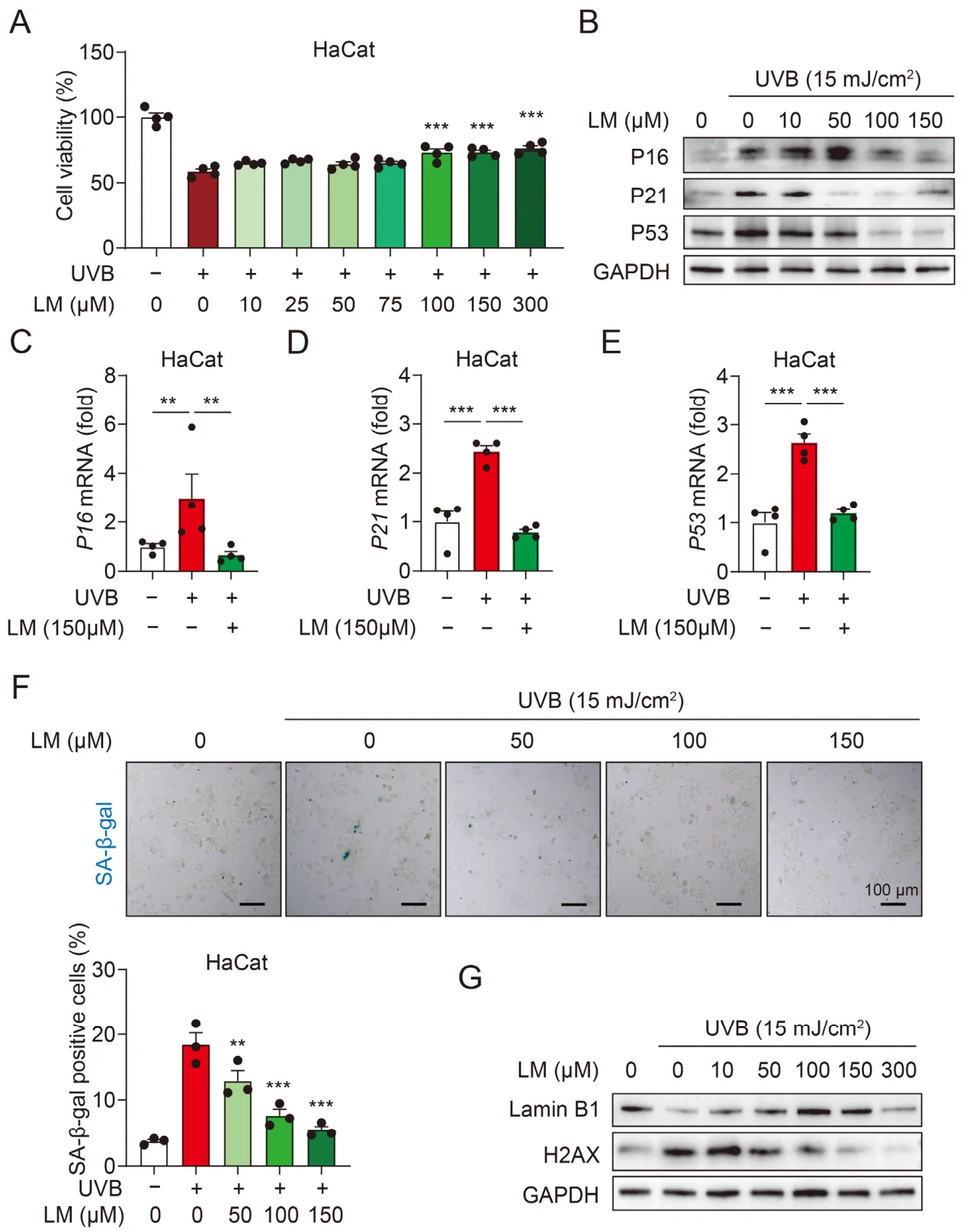
LM alleviates UVB-induced senescence and DNA damage in HaCaT cells. (**A**) Cell viability of HaCaT cells treated with gradient concentrations of LM after UVB irradiation (15 mJ/cm^2^) (*n* = 4). (**B**) Representative immunoblots of P16, P21, and P53 protein expression in UVB-irradiated HaCaT cells treated with increasing LM concentrations. (**C**–**E**) Relative expression levels of *P16* (**C**), *P21* (**D**), and *P53* (**E**) mRNA in HaCaT cells treated with or without 150 μM LM after UVB irradiation (*n* = 4). (**F**) Representative SA-β-gal staining images and quantification of SA-β-gal-positive cells in UVB-irradiated HaCaT cells treated with increasing LM concentrations (*n* = 3). (**G**) Representative immunoblots of Lamin B1 and γ-H2AX protein expression in UVB-irradiated HaCaT cells treated with increasing LM concentrations. Data are presented as mean ± SEM. ** *p* < 0.01, and *** *p* < 0.001.

Having established the photoaging model, we next evaluated the protective effects of LM. After UVB irradiation, HaCaT cells were treated with a concentration gradient of LM and incubated for 24 h; LM exhibited a statistically significant protective effect starting from 100 μM (Figure 2A). Following LM intervention, the expression of P16, P21, and P53 proteins was downregulated in a concentration-dependent manner, with the most significant inhibitory effect achieved at 150 μM (Figure 2B). Consistently, treatment with 150 μM LM significantly suppressed the UVB-induced elevation of *P16*, *P21*, and *P53* mRNA levels in HaCaT cells (Figure 2C–E). Furthermore, SA-β-gal staining demonstrated that the percentage of positive cells rose to approximately 20% after UVB irradiation and gradually declined with increasing LM concentrations; 150 μM LM treatment lowered the positive staining rate to a level close to that of the normal control group (Figure 2F).

Given that DNA damage is a key trigger of cellular senescence, we further evaluated the effect of LM on UVB-induced DNA damage by detecting the expression of Lamin B1 (a nuclear lamina protein and senescence biomarker) and γ-H2AX (a classical marker of DNA double-strand breaks) [30,31]. As depicted in Figure 2G, compared to the normal control group, the UVB model group exhibited a significant downregulation of Lamin B1 and a marked upregulation of γ-H2AX, indicating severe DNA damage. Notably, LM treatment gradually restored Lamin B1 expression and reduced γ-H2AX levels in a dose-dependent manner, indicating that LM effectively alleviates UVB-induced DNA damage in HaCaT cells. From these findings, we speculate that LM confers potent protection against UVB-induced senescence and DNA damage in HaCaT keratinocytes.

### 3.3. LM Inhibits UVB-Induced Extracellular Matrix Remodeling in HaCaT Cells

Skin photoaging is also characterized by aberrant extracellular matrix (ECM) remodeling, which underlies the clinical manifestations of wrinkles, loss of elasticity, and skin sagging [32]. To further elucidate the anti-photoaging mechanisms of LM, we investigated its regulatory effects on key molecules involved in ECM metabolism in UVB-irradiated HaCaT cells.

**Figure 3:**
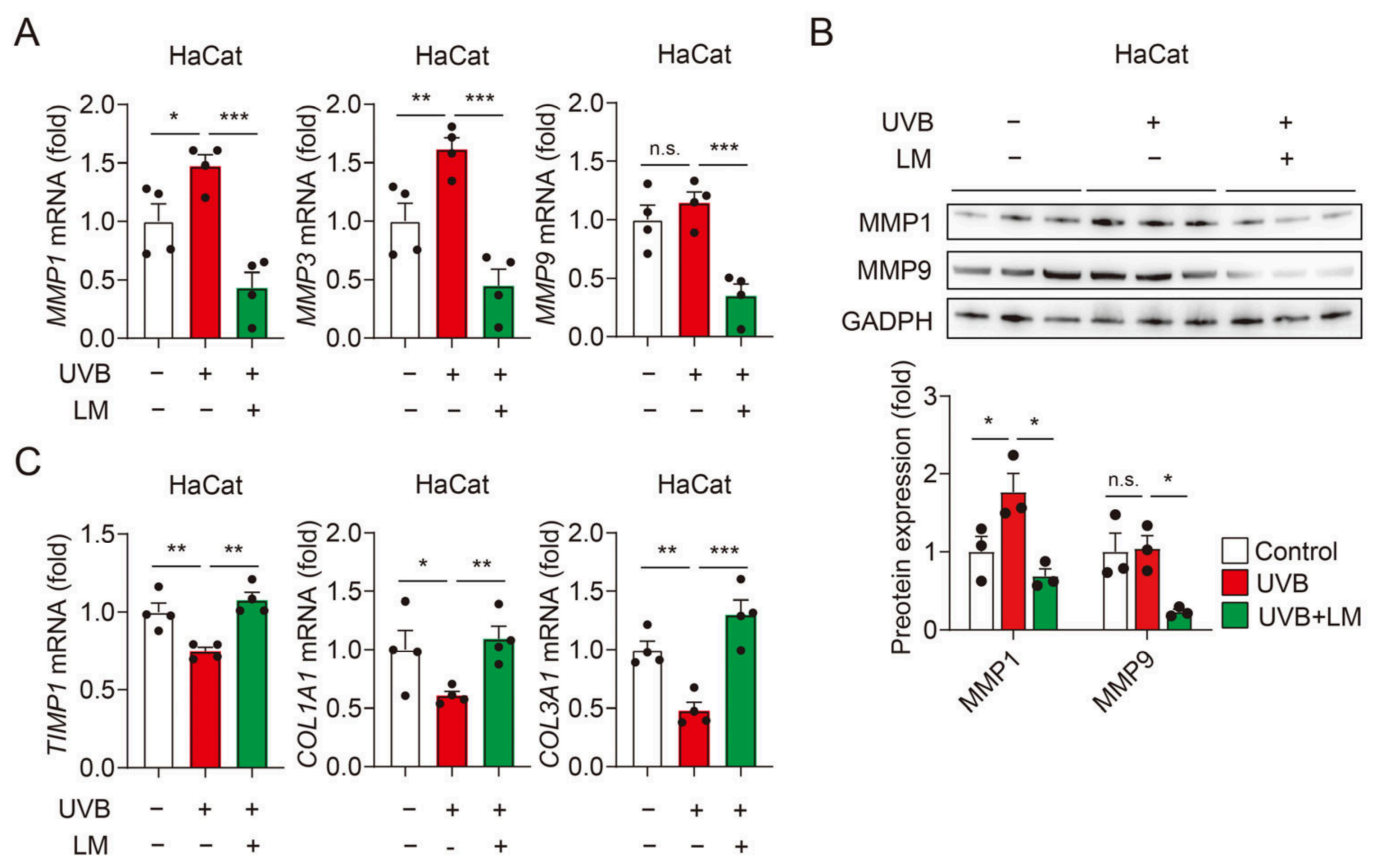
LM inhibits UVB-induced ECM remodeling in HaCaT cells. (**A**) Relative expression levels of *MMP1*, *MMP3*, and *MMP9* mRNA in HaCaT cells treated with or without LM (150 μM) after UVB irradiation (*n* = 4). (**B**) Representative immunoblots and quantification of MMP1 and MMP9 protein expression in HaCaT cells treated with or without LM (150 μM) after UVB irradiation (*n* = 3). (**C**) Relative expression levels of *COL1A1*, *COL3A1*, and *TIMP1* mRNA in HaCaT cells treated with or without LM (150 μM) after UVB irradiation (*n* = 4). Data are presented as mean ± SEM. * *p* < 0.05, ** *p* < 0.01, and *** *p* < 0.001; n.s., not significant.

Matrix metalloproteinases (MMPs) comprise a family of zinc-containing proteinases that are responsible for degrading collagen and elastic fibers [33]. Among them, MMP1 (encoding interstitial collagenase), MMP3 (Matrix Metalloproteinase 3), and MMP9 (encoding 92-kDa gelatinase) are strongly regulated by UV radiation in human skin [34]. LM treatment significantly reversed the elevated mRNA levels of MMP1, MMP3 and MMP9, as well as the elevated protein expression of MMP1 and MMP9, indicating that LM may inhibit excessive ECM degradation (Figure 3A,B). Tissue inhibitor of metalloproteinase 1 (TIMP1), the major endogenous inhibitor of MMPs, is essential for maintaining the dynamic balance between ECM synthesis and degradation [35]. Our results showed that UVB irradiation significantly downregulated *TIMP1* mRNA expression, whereas LM significantly restored *TIMP1* levels. Taken together with the changes in MMPs at both the mRNA and protein levels after LM treatment, these findings suggest that LM may help restore ECM homeostasis (Figure 3C).

Furthermore, we examined the effect of LM on collagen synthesis, the core structural component of the dermal ECM. UVB irradiation led to significant reduction in *COL1A1* (encoding type I collagen) and *COL3A1* (encoding type III collagen) mRNA expression, suggesting impaired collagen biosynthesis. LM intervention notably upregulated both *COL1A1* and *COL3A1* expression, suggesting that LM may modulate the expression of type I and type III collagen-related genes in HaCaT cells and reduce the levels of photoaging-related fibrotic markers (Figure 3C). Therefore, these results indicate that LM helps alleviate photoaging-related fibrotic changes by maintaining ECM integrity in UVB-irradiated HaCaT cells.

### 3.4. LM Attenuates UVB-Induced Photoaging in Skin Fibroblasts

**Figure 4:**
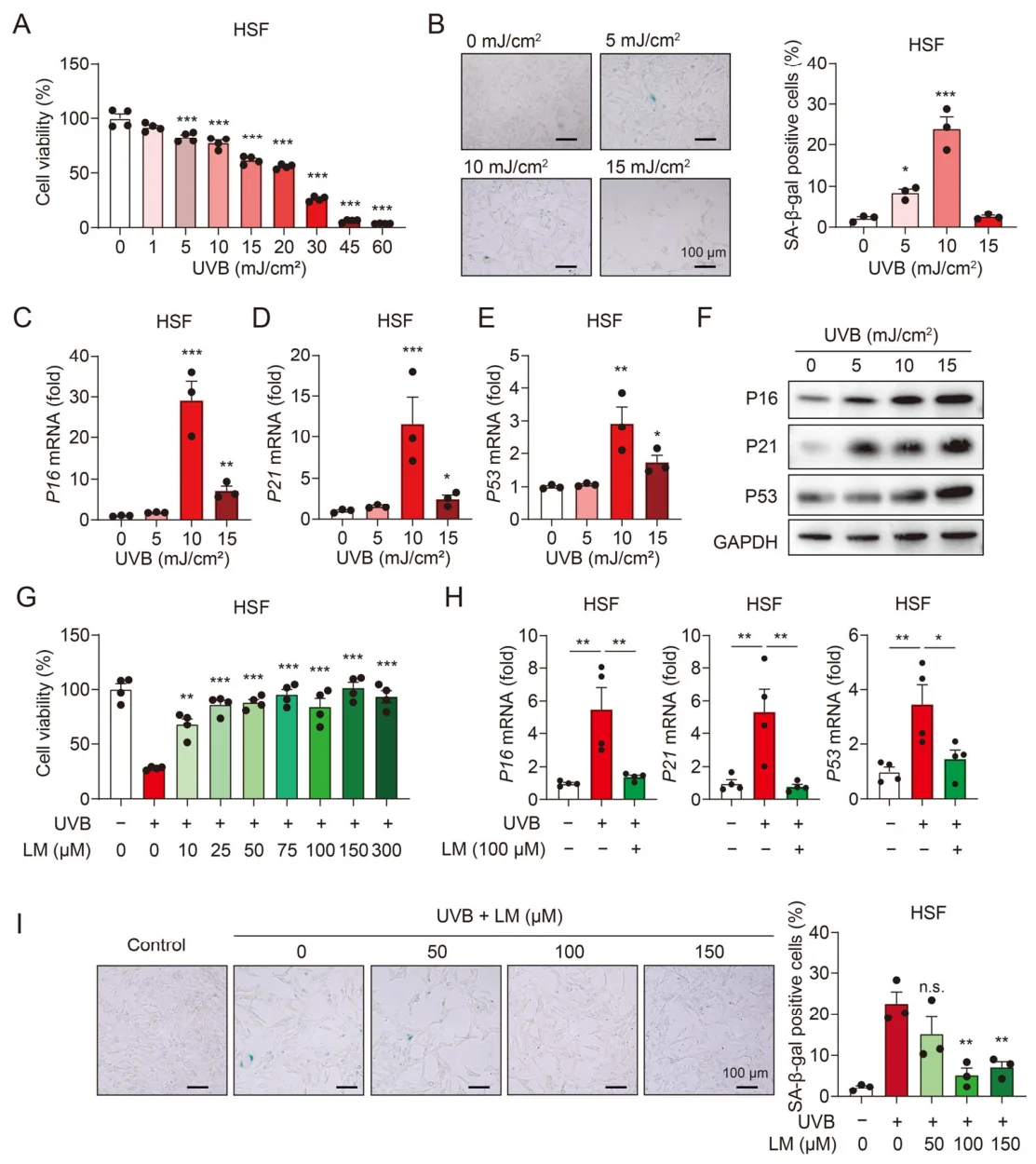
LM attenuates UVB-induced photoaging in skin fibroblasts. (**A**) Cell viability of HSF cells exposed to increasing doses of UVB irradiation (*n* = 4). (**B**) Representative images of SA-β-gal staining and quantification of SA-β-gal-positive cells at indicated UVB doses (*n* = 3). (**C**–**E**) Relative expression levels of *P16* (**C**), *P21* (**D**), and *P53* (**E**) mRNA in HSF cells exposed to increasing UVB doses (*n* = 3). (**F**) Representative immunoblots of P16, P21, and P53 protein levels in HSF cells following exposure to indicated UVB doses. (**G**) Cell viability of HSF cells treated with gradient concentrations of LM after UVB irradiation (10 mJ/cm^2^) (*n* = 4). (**H**) Relative expression levels of *P16*, *P21*, and *P53* mRNA in UVB-irradiated HSF cells treated with or without LM (100 μM) (*n* = 4). (**I**) Representative SA-β-gal staining images and quantification of SA-β-gal-positive cells in UVB-irradiated HSF cells treated with increasing LM concentrations (*n* = 3). Data are presented as mean ± SEM. * *p* < 0.05, ** *p* < 0.01, and *** *p* < 0.001; n.s., not significant.

To further validate the protective effects of LM across different skin cell types, we established a photoaging model in human skin fibroblasts (HSFs). After exposure to gradient doses of UVB, the viability of HSF cells decreased in a dose-dependent manner (Figure 4A). SA-β-gal staining confirmed the induction of premature senescence, and the most pronounced increase in positive staining was observed at 10 mJ/cm^2^ (Figure 4B). Concurrently, the mRNA levels of *P16*, *P21*, and *P53* were significantly upregulated, with expression peaking at 10 mJ/cm^2^ (Figure 4C–E), indicating successful model establishment at this optimal dose. Western blot analysis further confirmed that the protein levels of P16, P21 and P53 were elevated in a dose-dependent manner following UVB exposure (Figure 4F).

After 10 mJ/cm^2^ UVB irradiation, HSF cells were treated with gradient concentrations of LM. Notably, LM treatment at concentrations above 10 μM significantly restored cell viability, suggesting that HSF cells may be more sensitive to LM than HaCaT cells (Figure 4G). Furthermore, LM intervention substantially downregulated the expression of senescence-associated markers (Figure 4H and Figure S1) and reduced the percentage of SA-β-gal-positive cells (Figure 4I). Taken together, these results consistently demonstrate that LM protects HSF cells against UVB-induced photoaging.

### 3.5. LM May Act Through ME1/2-Dependent NADPH Regeneration to Inhibit Ferro-Aging in Skin Cells

**Figure 5:**
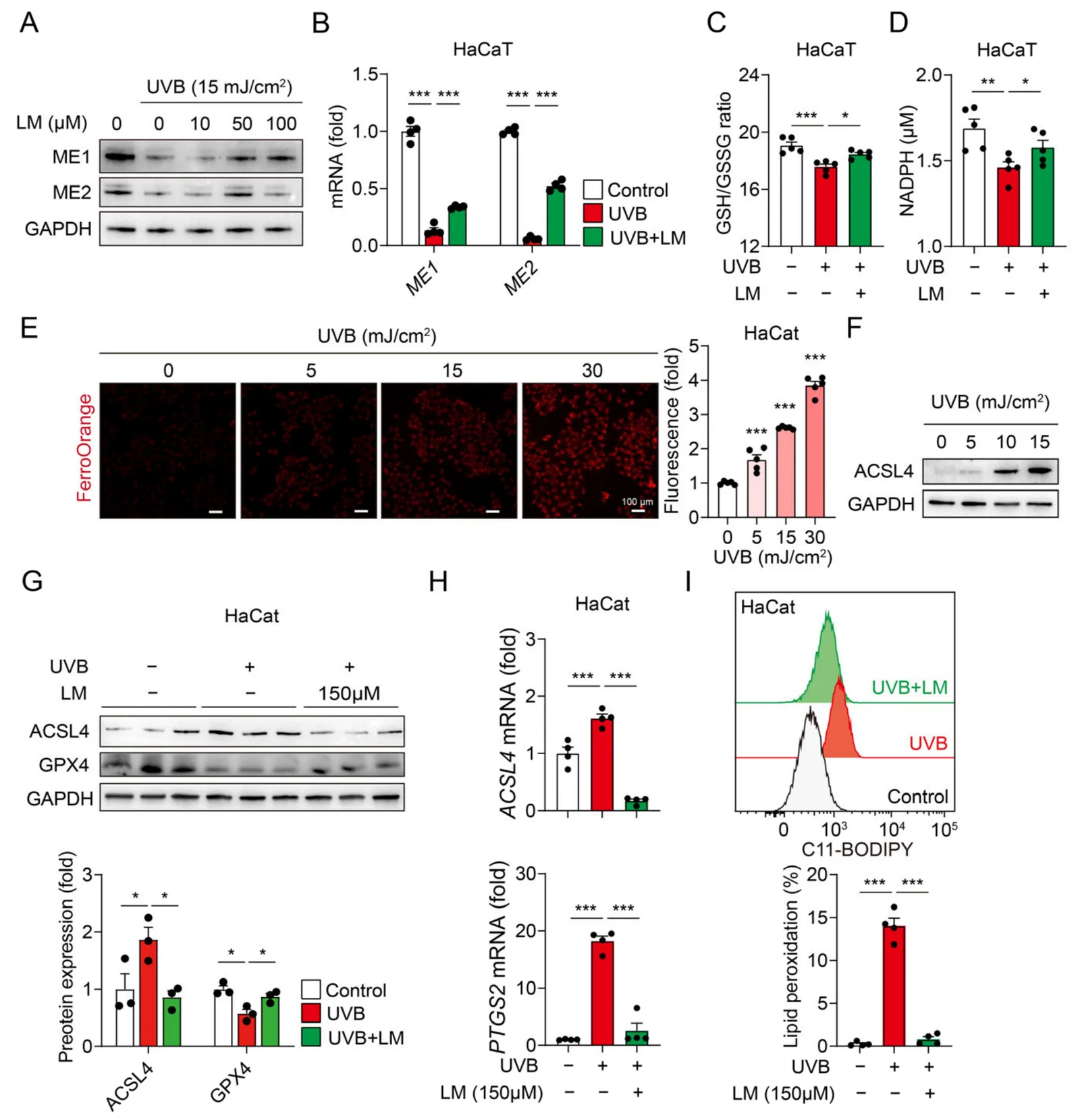
LM may act through the ME1/2-NADPH axis to inhibit ferro-aging in skin cells. (**A**) Representative immunoblots of ME1 and ME2 protein expression in UVB-irradiated HaCaT cells treated with increasing LM concentrations. (**B**) Relative expression levels of *ME1* and *ME2* mRNA in UVB-irradiated HaCaT cells treated with or without LM (150 μM) (*n* = 4). (**C**,**D**) Intracellular NADPH levels (**C**) and GSH/GSSG ratio (**D**) in UVB-irradiated HaCaT cells treated with or without LM (150 μM) (*n* = 5). (**E**) Representative images with quantification showing intracellular iron levels in HaCaT cells exposed to increasing UVB doses (*n* = 5). (**F**) Representative immunoblot of ACSL4 protein expression in HaCaT cells exposed to increasing UVB doses. (**G**) Representative immunoblots of ACSL4 and GPX4 protein expression in UVB-irradiated HaCaT cells treated with or without LM (150 μM) (*n* = 3). (**H**) Relative expression levels of *ACSL4* and *PTGS2* mRNA in UVB-irradiated HaCaT cells treated with or without LM (150 μM) (*n* = 4). (**I**) Lipid peroxidation levels in UVB-irradiated HaCaT cells treated with or without LM (150 μM) (*n* = 4). Data are presented as mean ± SEM. * *p* < 0.05, ** *p* < 0.01, and *** *p* < 0.001.

Having established the protective effects of LM against UVB-induced photoaging, we next sought to elucidate the underlying molecular mechanisms. Malic enzymes (ME1 in cytoplasm and ME2 in mitochondria) catalyze the oxidative decarboxylation of LM to generate NADPH, a critical reducing equivalent that maintains the GSH pool and supports antioxidant defenses [36]. As shown in Figure 5A,B, UVB irradiation significantly downregulated both the protein and mRNA levels of ME1 and ME2 in HaCaT cells, whereas LM treatment markedly restored their expression. Consistently, UVB exposure led to a substantial reduction in intracellular NADPH levels and a decline in the GSH/GSSG ratio, indicative of impaired redox homeostasis. LM intervention effectively antagonized these changes, restoring NADPH and the GSH/GSSG ratio to near-normal levels, suggesting that LM may exert its protective effects by maintaining the ME1/2–NADPH antioxidant axis (Figure 5C,D).

Ferro-aging is a recently identified form of cellular senescence characterized by iron accumulation and ACSL4-driven lipid peroxidation, but its role in skin photoaging remains largely unexplored [12]. We first examined whether UVB irradiation induces ferro-aging in HaCaT cells by measuring intracellular iron levels and ACSL4 expression. Our results showed that UVB irradiation induced a marked increase in intracellular labile iron pools, as detected by FerroOrange staining (Figure 5E), and dose-dependently upregulated ACSL4 protein expression (Figure 5F), suggesting the activation of ferro-aging. Notably, LM treatment significantly suppressed ACSL4 overexpression at both the protein and mRNA levels (Figure 5G,H and Figure S2). Furthermore, LM reversed the UVB-induced downregulation of GPX4 protein and upregulation of *PTGS*2 mRNA (Figure 5G,H and Figure S2), two well-established ferroptosis/ferro-aging markers. Consistent with these molecular changes, UVB-triggered lipid peroxidation, as assessed by C11-BODIPY fluorescence, was also significantly attenuated by LM treatment in both HaCaT and HSF cells (Figure 5I and Figure S2). Collectively, these findings suggest that the inhibitory effect of LM treatment on UVB-induced skin photoaging may be associated with the ME1/2–NADPH axis, thereby exerting a skin-protective effect.

### 3.6. LM Ameliorates UV-Induced Skin Photoaging In Vivo

**Figure 6:**
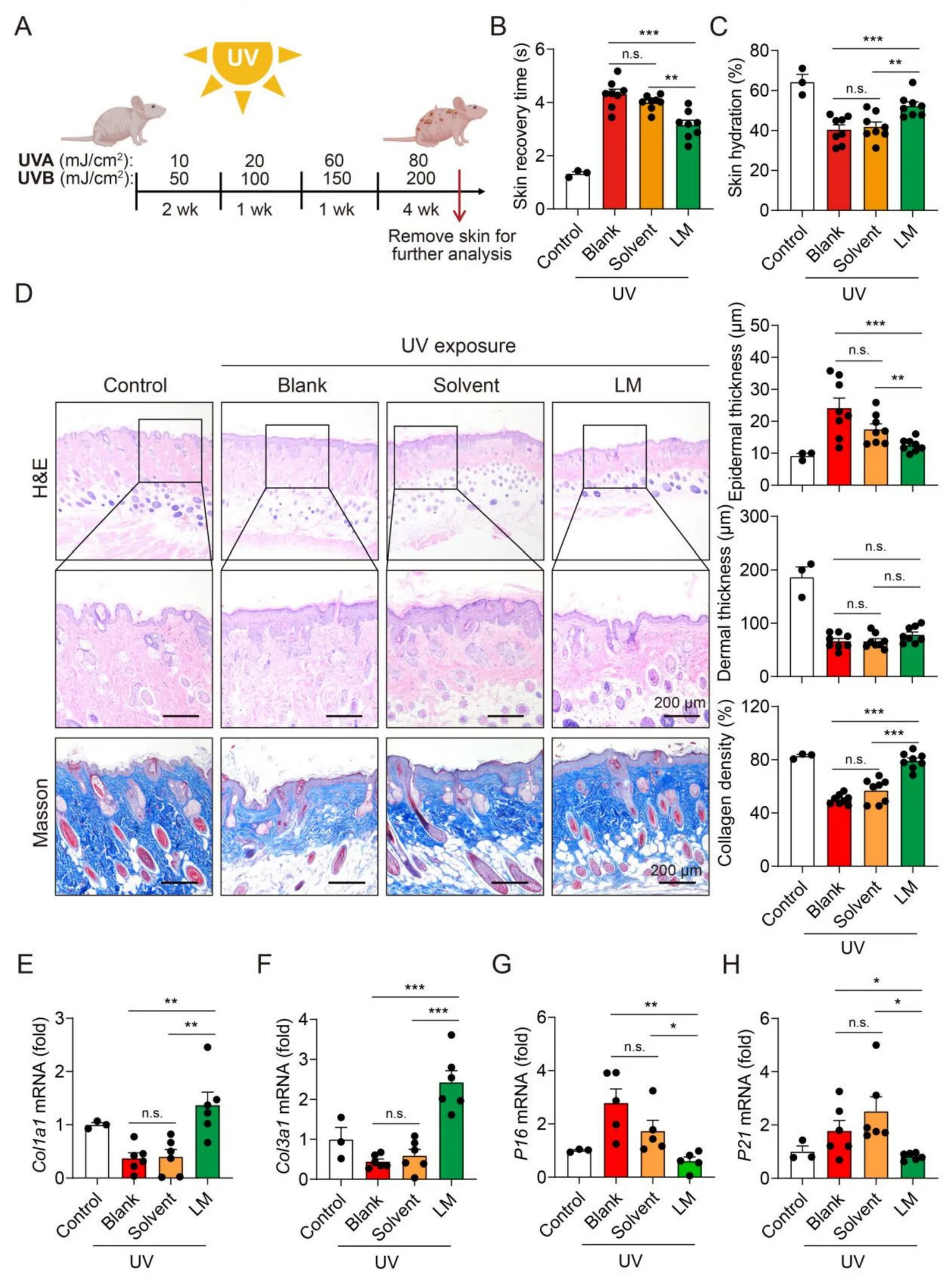
LM ameliorates UV-induced skin photoaging in vivo. (**A**) Schematic diagram of the chronic UV irradiation protocol and topical LM application in BALB/c nude mice. (**B**) Skin recovery time assessed by pinch test in mice from the indicated groups (Control, *n* = 3; Blank, Solvent, and LM, *n* = 8). (**C**) Skin water content in mice from the indicated groups (Control, *n* = 3; Blank, Solvent, and LM, *n* = 8). (**D**) Representative H&E and Masson’s trichrome staining images of dorsal skin sections, with quantification of epidermal thickness, dermal thickness, and collagen density (Control, *n* = 3; Blank, Solvent, and LM, *n* = 8). (**E**–**H**) Relative expression levels of *Col1a1* (**E**), *Col3a1* (**F**), *P16* (**G**), and *P21* (**H**) mRNA in mouse skin tissues from the indicated groups. For (**E**,**F**,**H**), Control, *n* = 3; Blank, Solvent, and LM, *n* = 6; for (**G**), Control, *n* = 3; Blank, Solvent, and LM, *n* = 5. Data are presented as mean ± SEM. * *p* < 0.05, ** *p* < 0.01, and *** *p* < 0.001; n.s., not significant.

To further evaluate the anti-photoaging efficacy of LM in a physiological context, we established a chronic skin photoaging model in BALB/c nude mice using a combined UVA/UVB irradiation protocol that more closely mimics natural solar radiation (Figure 6A) [37]. The photoaged model group exhibited a significantly prolonged skin recovery time as well as a reduced skin water content, indicating pronounced loss of skin elasticity and impairment of water-holding capacity. Notably, topical application of 10% LM, but not solvent, significantly shortened the recovery time and restored the skin hydration level (Figure 6B,C), demonstrating that LM effectively preserves skin elasticity and protects against UV-induced skin barrier dysfunction.

Photoaged skin is histologically characterized by epidermal hyperplasia, dermal atrophy, and collagen fiber disorganization. H&E and Masson’s trichrome staining was performed to evaluate histopathological changes. As shown in Figure 6D, chronic UV irradiation caused pronounced epidermal hyperplasia and dermal atrophy, along with fragmented and distributed collagen fibers. Although LM did not significantly reverse dermal atrophy, it significantly attenuated UV-induced epidermal hyperplasia and effectively preserved collagen content. Consistent with the histological findings, qPCR analysis revealed that UV irradiation significantly downregulated the mRNA expression of *Col1a1* and *Col3a1*, encoding type I and type III collagen, respectively (Figure 6E,F). Meanwhile, LM treatment reversed these phenomena, suggesting that LM treatment may restore the reduced collagen levels in vivo. Concurrently, LM treatment also markedly suppressed the upregulation of senescence-associated genes *P16* and *P21* (Figure 6G,H). Taken together, these in vivo results demonstrate that LM effectively protects against UV-induced skin photoaging, which is consistent with our in vitro observations.

### 3.7. LM Inhibits Ferro-Aging in UV-Irradiated Mouse Skin

**Figure 7:**
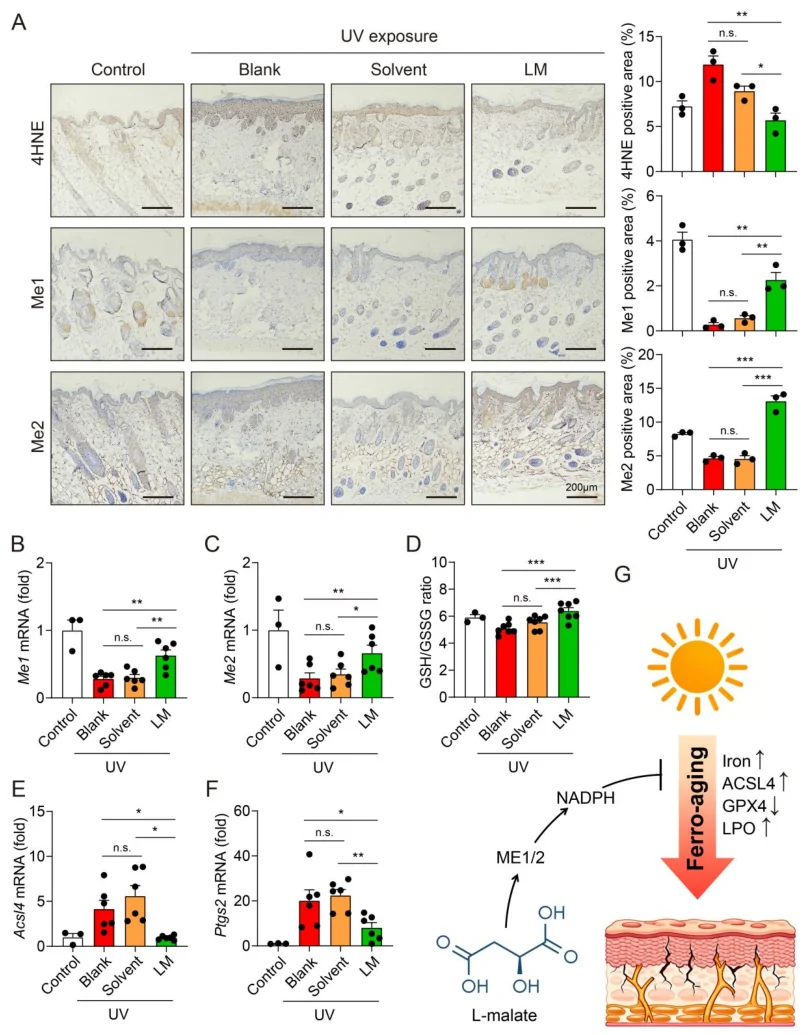
LM inhibits ferro-aging in UV-irradiated mouse skin. (**A**) Representative IHC staining images and quantification of 4-HNE, Me1, and Me2 in mouse dorsal skin sections from the indicated groups (*n* = 3). (**B**,**C**) Relative expression levels of *Me1* (**B**) and *Me2* (**C**) mRNA in mouse skin tissues from the indicated groups (Control, *n* = 3; Blank, Solvent, and LM, *n* = 8). (**D**) GSH/GSSG ratio in mouse skin tissues from the indicated groups (Control, *n* = 3; Blank, Solvent, and LM, *n* = 7). (**E**,**F**) Relative expression levels of *Acsl4* (**E**) and *Ptgs2* (**F**) mRNA in mouse skin tissues from the indicated groups (Control, *n* = 3; Blank, Solvent, and LM, *n* = 6). (**G**) Schematic diagram illustrating the proposed mechanism of LM against UV-induced skin photoaging. Data are presented as mean ± SEM. * *p* < 0.05, ** *p* < 0.01, and *** *p* < 0.001; n.s., not significant.

Then, we further investigated whether LM exerts its anti-photoaging activity in vivo by inhibiting lipid peroxidation and the ferro-aging cascade. 4-hydroxynonenal (4-HNE), a major and well-characterized end product of lipid peroxidation, is widely used as a reliable biomarker to evaluate oxidative lipid damage in tissues [38]. Immunohistochemical staining analysis revealed that chronic UV irradiation resulted in 4-HNE accumulation in the skin of mice compared with the normal control group. The solvent control group showed no significant difference in 4-HNE level compared with the UV model group. In contrast, LM intervention potently suppressed the UV-induced elevation of 4-HNE (Figure 7A), demonstrating that LM effectively inhibits cutaneous lipid peroxidation in vivo.

Consistent with our in vitro observations, LM treatment significantly restored the expression of Me1 and Me2 in UV-irradiated skin, which were markedly downregulated in the model group (Figure 7A,B). As a result, the GSH/GSSG ratio was significantly reduced by UV irradiation and was substantially restored by LM treatment (Figure 7D). We further examined the specific ferro-aging markers *Acsl4* and *Ptgs2* in mouse skin. As shown in Figure 7E, UV irradiation strongly upregulated *Acsl4* mRNA expression, which was significantly suppressed by LM treatment. Similarly, LM attenuated the UV-induced elevation of *Ptgs2* expression (Figure 7F). Collectively, these in vivo data suggest that LM may inhibit the ferro-aging cascade through the ME1/ME2 antioxidant axis, providing a mechanistic basis for its effects in vivo.

## 4. Discussion

In this study, we systematically investigated the protective effects of LM against UV-induced skin photoaging and elucidated its underlying molecular mechanisms both in vitro and in vivo. Our results demonstrate that LM effectively attenuates UVB-induced cellular senescence, DNA damage, and ECM degradation in HaCaT keratinocytes and HSFs. Mechanistically, we reveal that LM restores the expression of malic enzymes ME1 and ME2, which are downregulated by UVB exposure, thereby replenishing intracellular NADPH levels and preserving the GSH/GSSG redox balance. This metabolic restoration effectively blocks the ferro-aging cascade, as evidenced by the suppression of ACSL4 upregulation, GPX4 downregulation, and lipid peroxidation. Importantly, topical application of LM in a chronic UV-irradiated nude mouse model significantly ameliorated photoaging phenotypes, consistent with our in vitro observations (Figure 7G). These results not only provide experimental support for further exploring the feasibility of ferro-aging as a potential therapeutic target for skin photoaging, but also suggest the potential applications and development prospects of LM as a natural inhibitor of ferro-aging in the field of anti-aging skincare.

The quest for safe and effective natural products to combat skin photoaging has intensified in recent years. Plant-derived natural compounds, including polyphenols, flavonoids, carotenoids, and polysaccharides, have demonstrated considerable photoprotective and anti-aging activities through their antioxidant, anti-inflammatory, and ECM-protective properties [39,40]. However, many natural products face limitations including poor skin permeability, chemical instability, and the requirement for continuous exogenous reapplication due to their consumption during antioxidant reactions.

LM has potential advantages and a unique and favorable position among natural photoaging inhibitors. As an endogenous intermediate of TCA cycle and an FDA-approved food additive, LM is inherently biocompatible and safe for topical application. Unlike conventional antioxidants that scavenge ROS after their formation, this small-molecule organic acid helps improve the skin’s redox homeostasis by providing energy for the ME1/ME2-dependent endogenous NADPH production mechanism. The versatility of LM is further underscored by its ability to protect both keratinocytes and fibroblasts—the two principal cell types targeted in photoaging pathology—through a unified metabolic mechanism. Given that photoaging involves complex intercellular interactions between the epidermis and dermis [41], this cell-type-independent action may offer a notable advantage.

The central role of oxidative stress in UV-induced skin photoaging has been extensively documented in recent decades [42,43]. However, traditional antioxidant strategies targeting general ROS have shown limited clinical efficacy, suggesting that more specific oxidative damage pathways may be involved in the pathogenesis of photoaging. In 2012, Dixon et al. introduced an iron-dependent form of regulated cell death, which they termed ferroptosis [17]. However, ferroptosis represents an acute cell death program, while aging, characterized by gradual degenerative changes, are more likely driven by a sub-lethal iron-dependent process. Recent advances in aging research have identified ferro-aging as a conserved, iron-triggered, lipid peroxidation-driven cellular senescence program that is distinct from acute ferroptotic cell death [12]. Specifically, iron overload can catalyze the Fenton reaction to generate substantial amounts of ROS, thereby exacerbating cellular oxidative stress and causing cellular damage. ACSL4, as a key metabolic regulator, catalyzes the esterification of PUFAs, thereby rendering cells more susceptible to iron-mediated lipid peroxidation [21,22].

Because ferro-aging was only formally proposed this year, it currently lacks universally accepted diagnostic criteria, and no single marker can yet definitively distinguish it from related iron-dependent processes [23]. Operationally, we assessed ferro-aging as the convergence of three components, (i) iron-dependent lipid peroxidation, (ii) a senescence phenotype, and (iii) preserved cell viability, which discriminates this sub-lethal senescent state from acute ferroptotic cell death. It is important to emphasize that ferro-aging differs from general UV-induced oxidative stress—which is a broad, nonspecific ROS-mediated damage that is neither iron- nor ACSL4-dependent Similarly, transient UVB-induced DNA damage represents a trigger of senescence but does not itself constitute the iron-dependent, lipid peroxidation-driven program we observed. Future studies should focus on establishing standardized quantitative criteria and more specific biomarkers for ferro-aging, distinguishing it more rigorously from ferroptosis and general oxidative stress, and validating its relevance across different cell types, animal models, and ultimately human skin, so that ferro-aging can advance as a robust and actionable framework for promoting healthy aging.

The central role of oxidative stress in the aging process has also been reported [10]. Unlike ferro-aging, oxidative stress is a nonspecific, broad-spectrum cellular damage event that does not depend on iron accumulation or ACSL4 function. This suggests that future research on ferro-aging should focus on mechanistic dissection, marker screening and the establishment of a unified diagnostic framework for ferro-aging [44], translation of targeted interventions, and cautious clinical trials in human populations, so as to advance ferro-aging as a potentially promising strategy for promoting healthy aging in humans.

While iron accumulation in the skin has long been associated with accelerated aging [45,46], the specific contribution of ferro-aging to skin photoaging remained largely unexplored prior to our study. Our results demonstrate for the first time that UVB irradiation induces characteristic features of ferro-aging in skin cells, including increased intracellular labile iron pools, upregulated ACSL4 expression, and excessive lipid peroxidation. These findings fill a critical gap in our understanding of photoaging mechanisms and provide a new paradigm for developing targeted anti-photoaging interventions.

Although this study provides several important findings, it also has certain limitations that should be considered when interpreting the mechanistic conclusions. First, the proposed role of the ME1/ME2–NADPH axis is inferred from correlative evidence. We observed that LM treatment restores ME1/ME2 expression, NADPH levels, and the GSH/GSSG ratio, and that this is accompanied by suppressed ACSL4 upregulation and lipid peroxidation; however, these findings do not establish a causal or exclusive role for this axis. Second, because ME1/ME2-specific inhibitors, gene knockdown, and gene-overexpression tools were not employed in the current study, we were unable to functionally block or enhance ME1/ME2-mediated signaling. Consequently, we cannot exclude the involvement of other compensatory or parallel pathways (for example, the pentose phosphate pathway or isocitrate dehydrogenase-mediated NADPH regeneration) in mediating the protective effects of LM. Third, we measured ME1/ME2 mRNA and protein expression but did not directly assess their enzymatic activity or NADPH-producing flux; altered expression therefore does not necessarily equate to altered catalytic function. Future studies incorporating loss-of-function and gain-of-function approaches, together with direct enzyme-activity and metabolic-flux measurements, will be necessary to definitively establish the causal contribution of the ME1/ME2–NADPH axis to the anti-ferro-aging effects of LM.

In addition, young male BALB/c nude mice aged 6–8 weeks were selected for the in vivo experiments because mice at this stage maintain relatively good health, which facilitates the acquisition of stable and reliable data throughout the experimental period. Second, male mice do not exhibit the periodic fluctuations in sex hormones associated with the female estrous cycle, thereby reducing experimental variability caused by hormone cycle-induced fluctuations in baseline skin status. Moreover, because estrogen itself exerts photoprotective effects and male mice have lower estrogen levels than females, male nude mice were chosen for this study after comprehensive consideration. However, this choice did come at the cost of immune competence and sex/age representativeness, thereby limiting the generalizability and applicability of some findings to human populations. Future work will involve systematic cross-validation in immunocompetent models and across different sex and age groups.

In summary, this study provides compelling evidence that LM may protect against UV-induced skin photoaging by acting through the ME1/ME2-NADPH antioxidant axis and inhibiting the ferro-aging cascade. These findings not only advance our understanding of the molecular mechanisms underlying skin photoaging but also suggests the potential of LM as a novel, safe, and effective natural iron-mediated anti-aging agent with promising applications. Future research should focus on optimizing LM formulations for topical delivery, conducting human clinical trials to evaluate its efficacy and safety, and exploring its combination with other established photoprotective agents to open new avenues for comprehensive skin anti-aging therapy.

## 5. Conclusions

To our knowledge, this study is the first to show that UVB irradiation induces characteristic features of ferro-aging in skin cells, and it preliminarily explores the possible mechanism by which LM alleviates the ferro-aging cascade through the ME1/ME2–NADPH axis. Our study suggests that LM is a promising naturally derived candidate worthy of further investigation as a potential inhibitor of ferro-aging. Nevertheless, given that the present evidence is derived from immortalized cell lines and a small nude-mouse study, further preclinical and clinical studies are required before any translational or skincare applications can be claimed.

## Data Availability

The original contributions presented in this study are included in the article/Supplementary Materials. Further inquiries can be directed to the corresponding authors.

## Supplementary Materials

The following supporting information can be downloaded at: https://www.mdpi.com/article/10.3390/metabo16100775/s1, Figure S1. LM alleviates UVB-induced senescence and ECM remodeling in HSF cells. (A) Representative immunoblot of P53 protein expression in UVB-irradiated HSF cells treated with increasing LM concentrations. (B) Representative immunoblot of MMP1 protein expression in UVB-irradiated HSF cells treated with or without LM (100 μM). Figure S2. LM inhibits ferro-aging in HSF cells. (A) Representative images with quantification showing intracellular iron levels in HSF cells exposed to increasing UVB doses. (B) Representative immunoblots of ACSL4 and GPX4 protein expression in UVB-irradiated HSF cells treated with or without LM (100 μM). (C) Relative expression levels of *ACSL4* and *PTGS2* mRNA levels in UVB-irradiated HSF cells treated with or without LM (100 μM). (D) Lipid peroxidation levels in UVB-irradiated HSF cells treated with or without LM (100 μM). Data are presented as mean ± SEM. * *p* < 0.05, ** *p* < 0.01, *** *p* < 0.001.

## Abbreviations

4HNE, 4-hydroxynonenal; ACSL4, acyl-CoA synthetase long-chain family member 4; ECM, extracellular matrix; GPX4, glutathione peroxidase 4; GSH; reduced glutathione; GSSG, oxidized glutathione; LM, L-malate; ME1, malic enzyme 1; ME2, malic enzyme 2; MMP, matrix metalloproteinase; NADPH, nicotinamide adenine dinucleotide phosphate; PUFA, polyunsaturated fatty acid; ROS, reactive oxygen species; SA-*β*-gal, Senescence-associated-*β*-galactosidase; TIMP1, tissue inhibitor of metalloproteinase 1; TCA, tricarboxylic acid; UV, ultraviolet; UVB, ultraviolet B.

## References

[1] Mouawad J., Saadeh F., Tabosh H.A., Haddadin M.J., Gali-Muhtasib H. (2016). The photoprotective effects of 2-benzoyl-3-phenylquinoxaline 1,4-dioxide against UVB-induced damage in HaCaT cells. Med. Oncol..

[2] Liang J., Liu L., Tang H., Ma Q., Sang Y., Kang X. (2022). UVB-induced SFRP1 methylation potentiates skin damage by promoting cell apoptosis and DNA damage. Exp. Dermatol..

[3] Tobin D.J. (2017). Introduction to skin aging. J. Tissue Viability.

[4] Gu Y., Han J., Jiang C., Zhang Y. (2020). Biomarkers, oxidative stress and autophagy in skin aging. Ageing Res. Rev..

[5] Biniek K., Levi K., Dauskardt R.H. (2012). Solar UV radiation reduces the barrier function of human skin. Proc. Natl. Acad. Sci. USA.

[6] Kaltchenko M.V., Chien A.L. (2025). Photoaging: Current Concepts on Molecular Mechanisms, Prevention, and Treatment. Am. J. Clin. Dermatol..

[7] Rabe J.H., Mamelak A.J., McElgunn P.J., Morison W.L., Sauder D.N. (2006). Photoaging: Mechanisms and repair. J. Am. Acad. Dermatol..

[8] Zheng P., Kligman L.H. (1993). UVA-induced ultrastructural changes in hairless mouse skin: A comparison to UVB-induced damage. J. Investig. Dermatol..

[9] Bowden G.T. (2004). Prevention of non-melanoma skin cancer by targeting ultraviolet-B-light signalling. Nat. Rev. Cancer.

[10] Kammeyer A., Luiten R.M. (2015). Oxidation events and skin aging. Ageing Res. Rev..

[11] Kim D.J., Iwasaki A., Chien A.L., Kang S. (2022). UVB-mediated DNA damage induces matrix metalloproteinases to promote photoaging in an AhR- and SP1-dependent manner. JCI Insight.

[12] Liu L., Zheng Z., You W., Yang P., Wen Y., Qiao Y., Ma S., Zhang H., Zhang S., Xu G. (2026). Vitamin C inhibits ACSL4 to alleviate ferro-aging in primates. Cell Metab..

[13] Chrayteh D., Vaulont S., Peyssonnaux C. (2026). Iron-Skin Axis: Exploring the Interplay between Iron Homeostasis and Skin Disorders. J. Investig. Dermatol..

[14] Huang X., Alavi M., Chen L.C. (2026). A narrative review on critical roles of iron levels in skin tone, aging, and photoaging. Int. J. Women’s Dermatol..

[15] Maus M., López-Polo V., Mateo L., Lafarga M., Aguilera M., De Lama E., Meyer K., Sola A., Lopez-Martinez C., López-Alonso I. (2023). Iron accumulation drives fibrosis, senescence and the senescence-associated secretory phenotype. Nat. Metab..

[16] Yang Y., Zuo H., Ma X., Chen W., Sun M., Xiang Q., Wei Y., Zhao Y., Qi H., Liu T. (2026). Iron Overload-Induced Ferroptosis Drives Placental Dysfunction in Preeclampsia. Hypertension.

[17] Dixon S.J., Lemberg K.M., Lamprecht M.R., Skouta R., Zaitsev E.M., Gleason C.E., Patel D.N., Bauer A.J., Cantley A.M., Yang W.S. (2012). Ferroptosis: An iron-dependent form of nonapoptotic cell death. Cell.

[18] Du K., Wang L., Jun J.H., Dutta R.K., Maeso-Díaz R., Oh S.H., Ko D.C., Diehl A.M. (2024). Aging promotes metabolic dysfunction-associated steatotic liver disease by inducing ferroptotic stress. Nat. Aging.

[19] Mazhar M., Din A.U., Ali H., Yang G., Ren W., Wang L., Fan X., Yang S. (2021). Implication of ferroptosis in aging. Cell Death Discov..

[20] Liang S., Gao S., Wang F., Qu X., Kong L., Wang C., Li N. (2026). Ferro-aging: A potential driver of degenerative bone and joint diseases. Cell Commun. Signal..

[21] Doll S., Proneth B., Tyurina Y.Y., Panzilius E., Kobayashi S., Ingold I., Irmler M., Beckers J., Aichler M., Walch A. (2017). ACSL4 dictates ferroptosis sensitivity by shaping cellular lipid composition. Nat. Chem. Biol..

[22] Kagan V.E., Mao G., Qu F., Angeli J.P.F., Doll S., Croix C.S., Dar H.H., Liu B., Tyurin V.A., Ritov V.B. (2017). Oxidized arachidonic and adrenic PEs navigate cells to ferroptosis. Nat. Chem. Biol..

[23] Li C., Gong Z., Ye J., Ni Y. (2026). Vitamin C, ACSL4, and Ferro-Aging: Mechanistic Insights and Translational Perspectives from Primate Studies. Aging Dis..

[24] Lu J., Zhang S., Wu S., Gao C. (2025). l-malic acid: A multifunctional metabolite at the crossroads of redox signaling, microbial symbiosis, and therapeutic innovation. Arch. Biochem. Biophys..

[25] Fang X., Zhang J., Li Y., Song Y., Yu Y., Cai Z., Lian F., Yang J., Min J., Wang F. (2023). Malic Enzyme 1 as a Novel Anti-Ferroptotic Regulator in Hepatic Ischemia/Reperfusion Injury. Adv. Sci..

[26] Wu W., Lei R., Xu H., Lu L., Song C., Wu D., Li M., Chen L., Yu Y., Fang X. (2025). Malic enzyme 1 reverses cardiomyocyte senescence in a nitric oxide-dependent manner. Free Radic. Biol. Med..

[27] Lin L., Huang D., Huang H., Xie L., Huang Y., Ye C., Chu L., Qiao Y., Meng X., Cai S. (2025). Lung microbiota metabolite L-malic acid attenuates the airway inflammation in asthma by inhibiting ferroptosis. Toxicol. Appl. Pharmacol..

[28] Ding S., Yang Y., Mei J. (2016). Protective Effects of L-Malate against Myocardial Ischemia/Reperfusion Injury in Rats. Evid. Based Complement. Altern. Med..

[29] Jiang P., Du W., Mancuso A., Wellen K.E., Yang X. (2013). Reciprocal regulation of p53 and malic enzymes modulates metabolism and senescence. Nature.

[30] Shimi T., Butin-Israeli V., Adam S.A., Hamanaka R.B., Goldman A.E., Lucas C.A., Shumaker D.K., Kosak S.T., Chandel N.S., Goldman R.D. (2011). The role of nuclear lamin B1 in cell proliferation and senescence. Genes Dev..

[31] Mah L.J., El-Osta A., Karagiannis T.C. (2010). gammaH2AX: A sensitive molecular marker of DNA damage and repair. Leukemia.

[32] Quan T., Fisher G.J. (2015). Role of Age-Associated Alterations of the Dermal Extracellular Matrix Microenvironment in Human Skin Aging: A Mini-Review. Gerontology.

[33] Feng C., Chen X., Yin X., Jiang Y., Zhao C. (2024). Matrix Metalloproteinases on Skin Photoaging. J. Cosmet. Dermatol..

[34] Quan T., Qin Z., Xia W., Shao Y., Voorhees J.J., Fisher G.J. (2009). Matrix-degrading metalloproteinases in photoaging. J. Investig. Dermatol. Symp. Proc..

[35] Brew K., Nagase H. (2010). The tissue inhibitors of metalloproteinases (TIMPs): An ancient family with structural and functional diversity. Biochim. ET Biophys. Acta BBA Mol. Cell Res..

[36] Wang H., Cui W., Yue S., Zhu X., Li X., He L., Zhang M., Yang Y., Wei M., Wu H. (2024). Malic enzymes in cancer: Regulatory mechanisms, functions, and therapeutic implications. Redox Biol..

[37] Huynh C.B., Vu N.B., The Van T., Van Pham P. (2026). Establishment of a BALB/c Mouse Model for Photoaged Skin: Insights into UV-Induced Dermatological Changes. Ski. Res. Technol..

[38] Zhang X., Hou L., Guo Z., Wang G., Xu J., Zheng Z., Sun K., Guo F. (2023). Lipid peroxidation in osteoarthritis: Focusing on 4-hydroxynonenal, malondialdehyde, and ferroptosis. Cell Death Discov..

[39] Fernandes A., Rodrigues P.M., Pintado M., Tavaria F.K. (2023). A systematic review of natural products for skin applications: Targeting inflammation, wound healing, and photo-aging. Phytomedicine.

[40] Tanveer M.A., Rashid H., Tasduq S.A. (2023). Molecular basis of skin photoaging and therapeutic interventions by plant-derived natural product ingredients: A comprehensive review. Heliyon.

[41] Hu X., Chen M., Nawaz J., Duan X. (2024). Regulatory Mechanisms of Natural Active Ingredients and Compounds on Keratinocytes and Fibroblasts in Mitigating Skin Photoaging. Clin. Cosmet. Investig. Dermatol..

[42] Birch-Machin M.A., Bowman A. (2016). Oxidative stress and ageing. Br. J. Dermatol..

[43] Papaccio F., D’Arino A., Caputo S., Bellei B. (2022). Focus on the Contribution of Oxidative Stress in Skin Aging. Antioxidants.

[44] Gao S., Qi L., Bai W., Zhang J., Xu Z., Zhang Z., Li N., Liu W. (2026). Ferro-aging: A novel paradigm linking iron overload, lipid peroxidation and cellular senescence. Free Radic. Biol. Med..

[45] Leveque N., Robin S., Makki S., Muret P., Rougier A., Humbert P. (2003). Iron and ascorbic acid concentrations in human dermis with regard to age and body sites. Gerontology.

[46] Zhong J.L., Yiakouvaki A., Holley P., Tyrrell R.M., Pourzand C. (2004). Susceptibility of skin cells to UVA-induced necrotic cell death reflects the intracellular level of labile iron. J. Investig. Dermatol..

