# Supplementary material for: L-Malate Protects Skin Against Ultraviolet-Induced Ferro-Aging
Source: Metabolites. 2026 Oct 13;16(10):775. doi: 10.3390/metabo16100775 (PMC13707215; doi:10.3390/metabo16100775)
Supplement: Supplementary file 1 [file metabolites-16-00775-s001.zip › metabolites-4572534-supplementary.pdf]

L-malate Protects Skin against Ultraviolet-induced Ferro-aging

Xu *et al.*

***Leading corresponding author**

Xuexian Fang, Ph.D. Building No.7 in Shenyuan, School of Public Health, Hangzhou Normal University, 2318 Yuhangtang Road, Hangzhou 311121, China. E-mail: xfang@hznu.edu.cn.

This PDF file includes:

Figure S1

Figure S2

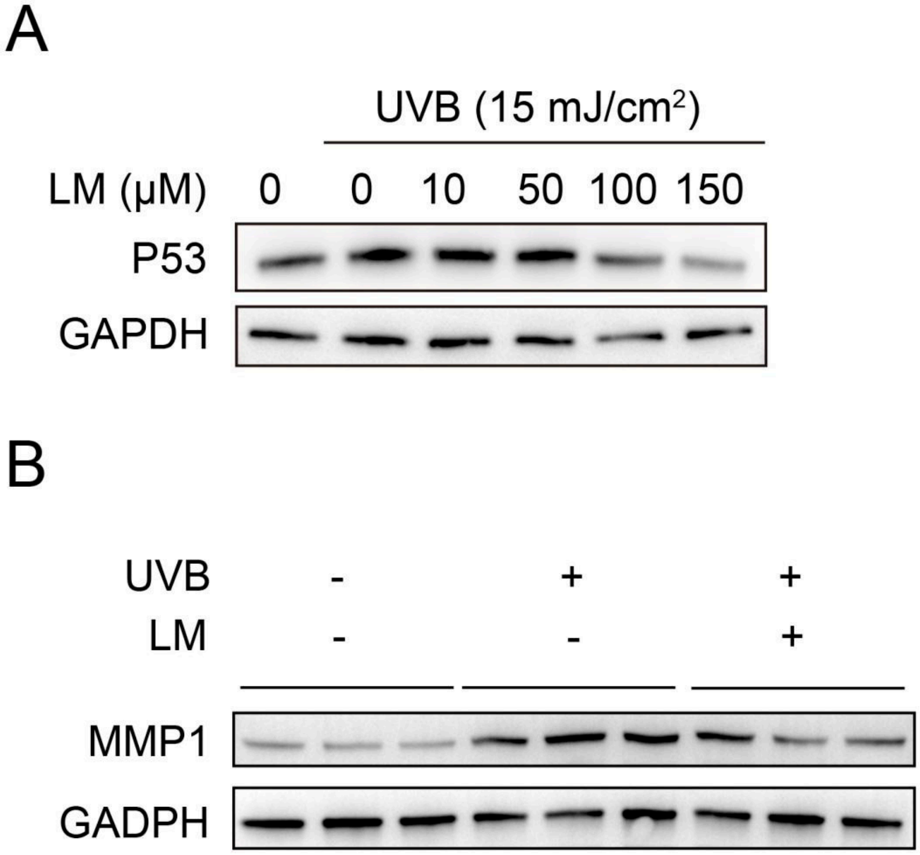

Figure S1. **LM alleviates UVB-induced senescence and ECM remodeling in HSF cells.**

(A) Representative immunoblot of P53 protein expression in UVB-irradiated HSF cells treated with increasing LM concentrations.

(B) Representative immunoblot of MMP1 protein expression in UVB-irradiated HSF cells treated with or without LM (100 μM).

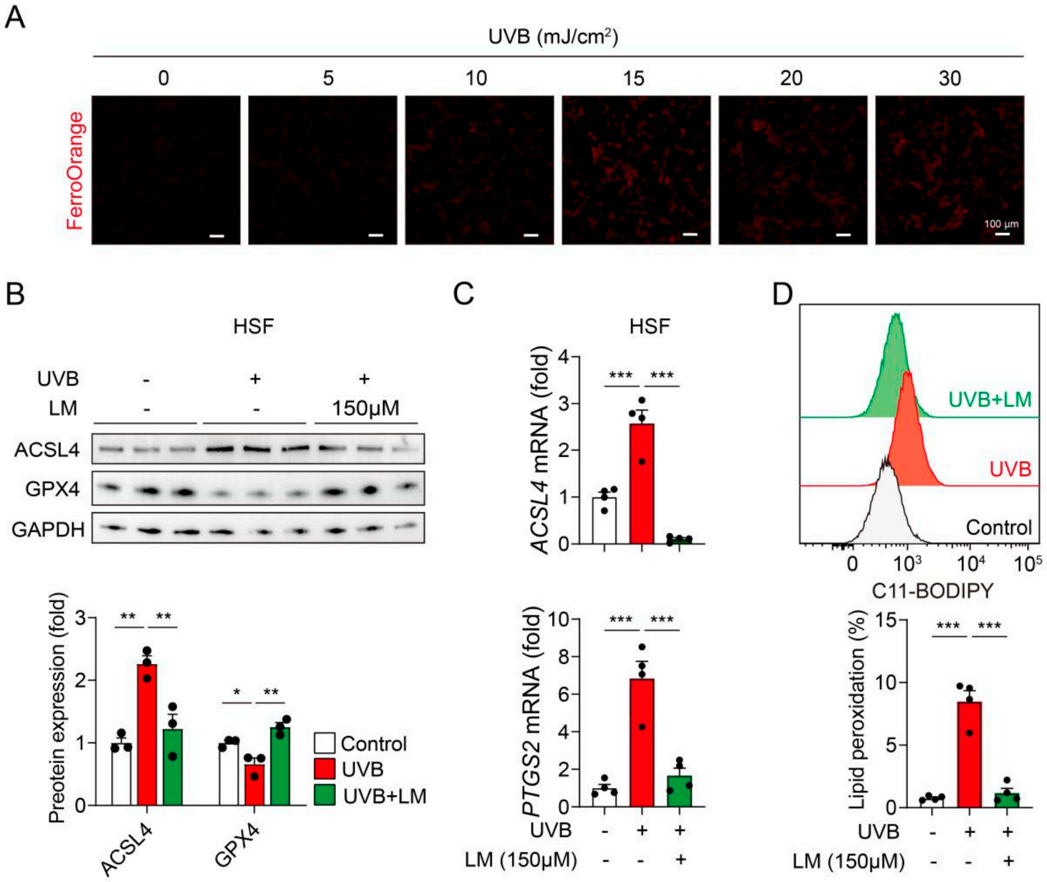

Figure S2. **LM inhibits ferro-aging in HSF cells..**

(A) Representative images with quantification showing intracellular iron levels in HSF cells exposed to increasing UVB doses.

(B) Representative immunoblots of ACSL4 and GPX4 protein expression in UVB-irradiated HSF cells treated with or without LM (100 µM).

(C) Relative expression levels of *ACSL4* and *PTGS2* mRNA levels in UVB-irradiated HSF cells treated with or without LM (100 µM).

(D) Lipid peroxidation levels in UVB-irradiated HSF cells treated with or without LM (100 µM).

Data are presented as mean ± SEM. *P<0.05, **P<0.01, ***P<0.001.
